# Are AI systems biased against the poor? A machine learning analysis using Word2Vec and GloVe embeddings

**DOI:** 10.1007/s00146-022-01494-z

**Published:** 2022-06-28

**Authors:** Georgina Curto, Mario Fernando Jojoa Acosta, Flavio Comim, Begoña Garcia-Zapirain

**Affiliations:** 1 Universitat Ramon Llull, IQS School of Management, Barcelona, Spain; 2 Universitat Autònoma de Barcelona, EINA Centre Universitari de Disseny i Art, Barcelona, Spain; 3 eVida Research Laboratory, University of Deusto, Bilbao, Spain

**Keywords:** Bias, Artificial intelligence, Embeddings, Poverty

## Abstract

Among the myriad of technical approaches and abstract guidelines proposed to the topic of AI bias, there has been an urgent call to translate the principle of fairness into the operational AI reality with the involvement of social sciences specialists to analyse the context of specific types of bias, since there is not a generalizable solution. This article offers an interdisciplinary contribution to the topic of AI and societal bias, in particular against the poor, providing a conceptual framework of the issue and a tailor-made model from which meaningful data are obtained using Natural Language Processing word vectors in pretrained Google Word2Vec, Twitter and Wikipedia GloVe word embeddings. The results of the study offer the first set of data that evidences the existence of bias against the poor and suggest that Google Word2vec shows a higher degree of bias when the terms are related to beliefs, whereas bias is higher in Twitter GloVe when the terms express behaviour. This article contributes to the body of work on bias, both from and AI and a social sciences perspective, by providing evidence of a transversal aggravating factor for historical types of discrimination. The evidence of bias against the poor also has important consequences in terms of human development, since it often leads to discrimination, which constitutes an obstacle for the effectiveness of poverty reduction policies.

## Introduction

It is widely documented that Artificial Intelligence (AI) reproduces and often amplifies biases against historically disempowered groups (Bolukbasi et al. 2016; Garga et al. 2018; Manzini et al. 2019; Nadeem et al. 2020). This constitutes a risk for the exacerbation of those biases offline and the eventual increase in discrimination (Vinuesa et al. 2020). AI systems are not ethically neutral but, more and more, we are all dependent on AI for our decisions (Fry 2018). In the information society, AI is at the core of high risk services, such as healthcare (Watson et al. 2019; Zetterholm et al. 2021; Vallès-Peris and Domènech 2021), financial services (Kostka 2019; Townson 2020; Lee and Floridi 2020; Aggarwal 2020; Anshari et al. 2021) justice and security (Poitras 2014; Hauge et al. 2016; Merler et al. 2019; Green et al. 2019) and even the military (de Vynck 2021). AI is also an integral part of marketing, predicting users’ interests through big data that contain each person’s personal digital profile, in what has been called “surveillance capitalism” (Zuboff 2019).

While the amount of algorithmic systems performing in questionable ethical manner continues to grow (Tsamados et al. 2021a), governmental efforts to regulate AI have gained momentum (Smith et al. 2016; SCMP Research 2020; European Commission 2021). At a regional level, the European Union is considered to have an ethically superior regulatory framework in terms of citizens’ rights (Allison and Schmidt 2019; Gill 2020; Imbrie et al. 2020; Roberts et al. 2021), which has a positive impact at a global level (Bradford 2020). At the core of the EU AI framework, there is the principle of “diversity, non-discrimination and fairness”, including the “avoidance of unfair bias”, especially in the case of the historically discriminated groups (HLEGAI 2019). However, the legal framework is not sufficient, considering that the ethical principles contained in the law are described as too abstract to implement in practice, often leading to some counterproductive practices, such as ethics shopping, ethics blue-washing, ethics lobbying, ethics dumping or ethics shirking (Floridi 2019a). There is a growing agreement on the urgent need to know how to translate this general ethical framework into the operational AI development (Floridi 2019b; Vakkuri et al. 2020; Morley et al. 2021a, b). In this context of “moral panic” (Ess 2020), there has been a proliferation of AI Ethics guidelines [more than 173 documents in existence in 2021 (Algorithm 2021)], there is a panoply of strategy proposals to detect and correct bias in the data of AI NLP systems (Bolukbasi et al. 2016; Garga et al. 2018; Manzini et al. 2019; Nadeem et al. 2020; Zhao et al. 2021), incipient attempts to train algorithms to detect bias (Sap et al. 2020; Jiang et al. 2021) and algorithmic mathematical constructs which try to achieve partial approximations to fairness (Dwork et al. 2011; Hardt et al. 2016; Kroll et al. 2017; Green and Hu 2018; Card and Smith 2020).

However, to translate the principle of AI fairness (HLEGAI 2019; European Commission 2021), into an operational reality, an in-depth analysis is required, far from the existing turmoil of quick-fix solutions. Bias within AI systems is only the tip of the iceberg, since AI reproduces the prejudices of the societies where they are trained (West et al. 2019; Vinuesa et al. 2020) in an unsupervised manner (Radford et al. 2019; Talmor et al. 2021), either within the data (Rudinger et al. 2018; Chiappa et al. 2020), the algorithms (Mittelstadt et al. 2016; Tsamados et al. 2021b) or even as a result of development procedures (Floridi 2019a; Vakkuri et al. 2020). Therefore, trying to solve the AI ethical problems only through a technical approach is clearly insufficient, since it only has a superficial impact on fundamental inequalities (Zajko 2021). Blodgett et al. (2020) analysed 146 papers studying bias in NLP systems (published prior to May 2020) and concluded that these papers do not provide an actual conceptualisation of bias outside NLP systems. Card and Smith (2020) suggest that literature on fairness within ML depends mostly on assumptions. A growing number of voices highlight the need for involvement from the social sciences perspective (Green and Hu 2018; By et al. 2019; Kusner and Loftus 2020; Zajko 2021) since bias needs to be discussed in the “onlife”, using Floridi (2015). In fact, the aim to debias AI systems is based on the illusion that there is a neutral value-free environment, when it is really meant to align with the dominant scientific, social and political values (Green 2020).

When we analyse the nature of bias, it becomes evident that we cannot draw a hard line between what is sufficient and insufficient proof of it, since it is based on our beliefs and a characteristic of human cognition (Allport 1954; Reicher 2007; Pettigrew 2020; Paolini et al. 2021). In fact, the reason why human beings are not only perceived based on their individual characteristics is because we do not have enough time to understand every single detail of every person. Therefore, we put information into categories and generalise based on previous experience. Overgeneralised and erroneous beliefs lead to prejudices. When prejudices have a social category, they are described as stereotypes and, when they are transmitted through the linguistic process, we know them as bias, generating a self-perpetuating cycle in which prejudices are socially shared and maintained (Maass 1999; Beukeboom and Burgers 2019). Where bias is the linguistic expression of shared social prejudices within a specific culture, discrimination has been defined as an action of exclusion as a result of prejudice (Allport 1954).

But seeing the tip of the iceberg (bias in AI systems), also tells us that there is an iceberg. Bias in AI acts as a mirror, showing the prejudices that go unnoticed off-line and helping us to evidence an unnoticed discriminatory phenomenon (Hoffmann 2019). While algorithms reproduce inherent tensions at a technical level (Hacker 2018), these data can be used as a warning towards a stigma, which can then be studied from a social sciences perspective since it has a history behind (Zajko 2021). This is precisely what this paper offers: the evidence of bias against the poor in social networks, a neglected type of discrimination in both AI bias and social sciences literature, named “aporophobia” by the philosopher Adela Cortina (2017).

The bias against the poor, which often leads to discriminatory behaviour, has dramatic repercussions since it hinders the effective implementation of poverty reduction policies (Arneson 1997; Applebaum 2001; Everatt 2009; Nunn and Biressi 2009), hampering the work towards the first Sustainable Development Goal of the United Nations (no poverty). It also has a clear impact on the historically discriminated groups (Alessina and Glaeser 2013) and it is closely related to gender discrimination in capitalist development (Folbre 2021). Sadly, it has been underestimated as a transversal type of discrimination, since there is the tendency within the antidiscrimination discourse towards a single-axis thinking (Crenshaw 1991). However, stereotypes exist within a network of beliefs (Freeman and Ambady 2011), where there is a dynamic interaction among them (Ridgeway and Smith-Lovin 1999) and an aggravating effect for what Hoffman defines as the “multi-oppressed” (2019).

Eubanks (2018) identifies algorithms that discriminate the poor and O’Neal (2016) describes how some predatory AI systems target people in need. However, there is no evidence about bias against the poor in the existing literature. This study aims to fill in that gap by offering a first approach to the identification and measurement of bias against the poor in the publicly available Google News Word2, Wikipedia GloVe and Twitter GloVe pre-trained word embeddings, providing a study at scale and in context (Joseph and Morgan 2020).

This article offers an interdisciplinary contribution to the topic of AI and societal bias, in particular against the poor, and it is organised in 5 parts. First, it provides an analysis on the roots of discrimination against the poor. Then, we present the materials and methods being used, such as the rationale behind the target terms and attributes that are being searched, the pre-trained word embeddings that have been analysed and the methodology to identify and measure bias against the poor using Natural Language Processing (NLP). The key results are then analysed to discuss the main implications and conclude.

## The roots and consequences of bias against the poor

Redistributive justice is at the very foundation of welfare states, where the principle of equal opportunity is considered to be the main political answer to reduce poverty and an attempt to promote social mobility. But the rhetoric of equal opportunity has also been associated with the blamefulness of the poor, who are considered responsible for not climbing up the social ladder (Young 1964; Anderson 1999; Sandel 2020). However, meritocracy, understood as a system where you prosper by working hard, is more collective entelechy than a reality: only 7% of the population of the United States within the 20% lower rents get to the 20% top rents in their lifetime (Chetty et al. 2014) and some European countries, such as Germany, have lower social mobility than the US (OECD 2018). In fact, the principle of equal opportunity, per se, can be considered an ideal, since every individual is inevitably exposed to different environments from the moment of birth (Fishkin 2014). This shared belief, though, assigns the responsibility to avoid poverty to each individual, promoting a competition among citizens seeking to work their way up and obtain social recognition (Fraser and Honneth 2003; Mounk 2017) especially in the US, where citizens overestimate the real possibilities to climb up the ladder, as opposed to the Europeans, who tend to underestimate their possibilities of social mobility (Alesina et al. 2018). In the meritocratic logic, where technocratic governments are mainly oriented towards the market, the rich are considered to be the winners, associated with being hard-working and smart, while the poor are considered also to deserve their fate (Mounk 2017; Sandel 2020). The disempowerment and resentment of the poor are aggravated by the increasing inequality in the US since 1980s (Piketty et al. 2018), which has boosted as a result of the COVID-19 crisis, according to Gini coefficient estimates.

The bias against the poor, therefore, is aggravated by the blamefulness associated to this condition and leads to discrimination. This has an impact at a macro-international level, where developing countries are considered to be responsible for their poverty, instead of working towards farer deals in areas, such as international commerce and financial markets (Sampedro 1972; Tortosa 2001; Yapa 2002; Lamo de Espinosa 2004; Reis et al. 2005). At a meso-national level, discrimination towards the poor constitute a hindrance for the effective implementation of poverty reduction policies (Arneson 1997; Applebaum 2001; Everatt 2009; Nunn and Biressi 2009), where policy-makers are forced to justify which poor are victims of bad luck, and therefore deserving support, and which are deserving aid (“luck egalitarism”) (Anderson 1999). Finally, at a micro-personal level, the stigma towards the poor generates a self-depreciation, which contributes to a self-fulfilling prophecy of failure to climb up the ladder (Honneth 1996; Habermas 1990; Taylor 1931). Nevertheless, bias against the poor reflects a morally narrow view of social merit, limited to economic and professional credentialism. It is only when the focus is on salary and consumption that badly paid jobs lack social recognition. During the COVID-19 crisis, precariously paid workers in sectors, such as delivery and hospital staff enjoyed an increased social recognition, which is essential to overcome the feelings of shame among the stigmatised and beliefs of deservingness on the side of the stigmatisers. (Goffman 1963; Hegel 1991; Honneth 1996).

By offering preliminary evidence about the bias against the poor, this study only scratches the surface of a global and transversal type of social exclusion that potentially can affect 700 M people (10% of the total world population) that currently live in extreme poverty, according to the United Nations (evidence suggests that global poverty could increase by 8% as a result of COVID-19) and is not limited to developing countries (in 2019, 92,4 M people in the EU-27 are at risk of poverty or social exclusion (21.1% of EU-27 population) according to Eurostat).

## Detection of bias against the poor: materials and methods

### Materials

#### Target terms and attributes

Bias cannot be treated as a generalizable manner, but in a context (Zajko 2021), for which a framework is required, from the social sciences perspective, to obtain and analyse meaningful data that can be offered by AI. With that purpose, this paper offers a model to identify and interpret bias based on Cortina’s work on aporophobia (rejection towards the poor) (2017) and Allport’s categorization of the degrees of “negative action” associated with prejudices (1954).

Cortina uses a list of 17 expressions associated with rejection towards the poor. In our study, we have used 262 synonyms, antonyms and related terms to Cortina’s expressions to understand how these are related to the concepts of “rich” and “poor”. We investigate whether or not a set of favourable attributes is closer or not to the target term “rich” (positive bias towards the rich) and a set of unfavourable attributes more closely related or not to the target term “poor” (bias against the poor).

This preliminary approach to measure bias against the poor offers some limitations due to the polysemy of the terms “rich” and “poor”. The term poor carries a negative sentiment in English which is not limited to socio-economic topics and the opposite happens with the term “rich”. One can talk, for example, about poor results or poor language, which surely has no direct relation to poverty, described as the lack of freedom to carry out a meaningful life with dignity (Sen 2001). Used as adjectives, the terms “rich” and “poor” can be associated to positive and negative attributes for reasons that might have no direct connection to bias against poor people. Therefore the obtained results need to be considered with caution. Further studies using a larger list of key terms related to poverty which do not offer polysemy (such as unemployed or homeless) should be carried out to contrast the results.

However, one should also carefully analyse why such a negative sentiment is associated with the adjective “poor” while there is a positive connotation of the adjective “rich”, as it is the case with other existing types of bias in terms of race, for example, (where implicit positive connotations are associated with the term “white” as opposed to negative implicit connotations to the term “black”, as shown in the Harvard Implicit Association Test) (Xu et al. 2014). Further studies should also analyse the origin of the negative connotations associated to the term poor.

Following Allport’s categorization of “negative action” resulting from prejudices, the favourable and unfavourable attributes for which the association is measured with the target terms “rich” and “poor” are grouped into 1 first category expressing “belief” (28 favourable and 23 unfavourable words) and 5 categories expressing different degrees of favourable (93 words) or unfavourable attitudes (119 words). The different categories defined by Allport are not sealed compartments, but a conceptual way to organize the favourable and unfavourable expressions that are part of the study and can potentially express bias against or in favour of the poor and the rich.

#### Word coding/embeddings

We have measured the semantic distance between the 262 favourable- and unfavourable attributes-related Cortina’s expressions and the key terms “rich” and “poor” using vector word representations, which is the state-of-the-art technique in natural language processing. More specifically, we have observed the semantic relationships between the vector word representations in word embeddings (key terms and attributes) in a simple and intuitive way using the cosine distance. In our model, we have proposed the use of three types of categories of words, which we have called favourable, neutral and unfavourable attributes, to measure the semantic distance to the key terms “rich” and “poor” to detect and measure bias.

The concept of embedding was born as dense vector representations of words or sentences, with the ability to map, syntactic and semantic relations in a vector space, which is core to Natural Language Processing (NLP) application (Almeida and Xexéo 2019; Camacho-Collados and Pilehvar 2020). Word embeddings are classically classified into two types: count-based embeddings, whose representation is derived from word counts and word frequencies, and predict-based embeddings, which are derived from word context (words neighbouring a core word). The latter are the base of cutting-edge Neural Language Models approach (Adamuthe 2020). The most used embeddings are the predict-based family (Gutiérrez and Keith 2019). For our work, we have used Word2Vec (Mikolov et al. 2013a), FastText (Bojanowski et al. 2016) and Glove (Pennington et al. 2014) which are unsupervised approaches based on the hypothesis that words whose occurrence arises in the same contexts tend to have similar meanings. Using this approach for our work, we are able to measure the distance between words/vectors within a context, since the embedding contains the context information of the data used to build it.

The technique we present in this paper could be compared, in a certain way, with a text mining analysis based on an exploratory study where word counting and word clouds could be proposed for a semantic analysis, where the word with the highest frequency is considered the most relevant. However, for a study involving millions of different grammars, the task would become very complex to reach relevant conclusions in terms of identifying bias. Besides, we have selected to perform a vectorial study of the numerical representations of the embedding context, because it offers better explainability, required for all approaches based on machine learning models.

#### Pre-trained embeddings

We have detected and measured bias against the poor in pre-trained word embeddings, which are trained on large datasets and constitute an appropriate and available option to measure the distance between the target terms and attributes of the study. In future studies, we aim at training our own embedding, which will allow us to ensure the quality of the data involved and to have more control on the amount of context being compared, providing the possibility, for example, to look for bias against the poor not only using term associations, but also sentence associations, which would contribute to solve the polysemy caveat of the terms “rich” and “poor” identified in this study.

We have obtained results from three different embeddings (Google News Word2, Wikipedia GloVe and Twitter GloVe). We have then compared the results obtained, reaching conclusions about the common trends among the three datasets as regards bias against the poor and also about the specificities of this phenomenon in each embedding.

- **Google news word2vec pre-trained embedding**
  The Google News 300 word embedding is a pre-trained model of word representation as vectors, using 300 features or coordinates in a 300-dimensional system. This model was trained using a Google News database (about 100 million words). A representation of more than 3 million words and phrases was obtained. The base algorithm used for the creation of this embedding was proposed by Mikolov et al. (2013). The resulting model has a weight of 1.3 Gb.
- **Wikipedia GloVe pre-trained embedding**
  The Wikipedia GloVe word embedding is a pre-trained word representation model, using the GloVe technique based on the global co-occurrence matrix between words. The training corpus is a dataset of Wikipedia publications. The Wikipedia corpus contains about 2000 million words of text from 4400 million Wikipedia pages consolidated up to 2014. Additionally, it contains the Gigaword 5 dataset, a comprehensive collection of news text data that has been acquired over several years by the Linguistic Data Consortium (LDC) and contains 4 billion words. The resulting word representation model contains 6 billion tokens, 400 thousand vocabulary words and was trained with all words uncased. Thus, there are four versions of trained embeddings with different vector dimensions: 50, 100, 200 and 300 dimensions. The weight of the resulting model is 822 MB.
- **Twitter GloVe pre-trained embedding**
  The Twitter GloVe word embedding is a pre-trained word representation model using the GloVe technique based on the global co-occurrence matrix between words. The training corpus is a dataset of tweets extracted from Twitter social network. For the construction of the model, 2 billion tweets written in English were taken. The resulting model contains 27 billion tokens, 1.2 million vocabulary words and was trained with all words uncased. For this word representation model, there are 25-, 50-, 100- and 200-dimensional versions. The weight of the resulting model is 1.42 GB

### Methods

**Fig. 1:**
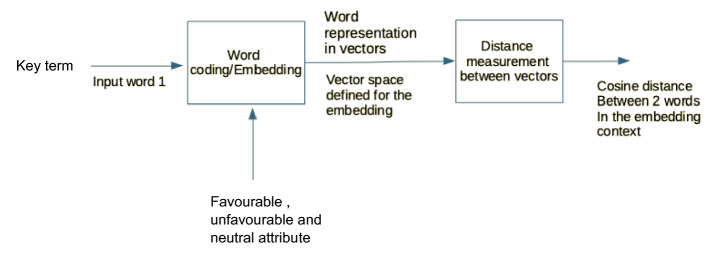
Block diagram of the proposed solution

The following diagram (Fig. 1) illustrates the proposed solution to detect and measure bias against the poor using the key terms “rich” and “poor”, 262 “favourable” and “unfavourable” attributes and vector word representations to measure semantic proximity using the cosine distance in pre-trained word embeddings (Google News Word2Vec, Wikipedia GloVe and Twitter GloVe). We have also tested the model using “neutral” attributes. We are fully aware of the limitations attached also to the use of some of these attributes, in particular those that work both as nouns and adjectives. For this reason, a rich array of expressions was chosen.

#### Semantic analysis of words based on vector distances

The basis of this work is the semantic analysis based on distance. To get reliable information of the relationship between words, we have decided to use the cosine distance, since this numeric metric preserves the relative direction of two vectors, inside the vectorial space (in our case, the meaning direction between words).

#### Cosine distance between words

The cosine of angle indicates directly proportional similarity between two-word vectors. As the metric increases, it indicates that there is greater similarity between the words. Mathematically, similarity between vectors is defined as the cosine of the angle between the vectors, so the closer the vectors form an angle to zero, the more similar they are. The cosine of the angle is defined with Eq. (1):

1 $${\varvec{c}}{\varvec{o}}{\varvec{s}}({\varvec{\theta}})=\frac{{{\varvec{A}}}^{{\varvec{T}}}{\varvec{B}}}{|{\varvec{A}}|\cdot |{\varvec{B}}|}$$

Thus, the cosine of the angle is defined as the dot product divided by the multiplication of its norms.

#### Calculation of the dot product between words

The similarity metric based on the dot product between the word vectors is directly proportional to the scalar value resulting from the operation. However, this metric increases not only by the cosine of the angle of the vectors, but also by the length of the vectors, so it is necessary to take into account that the metric may be biased by the length of the word vectors. The dot product is defined as in Eq. (2):

2 $${{\varvec{a}}}_{1}{{\varvec{b}}}_{1}+{{\varvec{a}}}_{2}{{\varvec{b}}}_{2}+\dots {+{\varvec{a}}}_{{\varvec{n}}}{{\varvec{b}}}_{{\varvec{n}}}=|{\varvec{A}}||{\varvec{B}}|{\varvec{c}}{\varvec{o}}{\varvec{s}}({\varvec{\theta}})$$

#### Semantic relations between target and attribute words based on cosine distance

262 registers were built to capture the semantic relationships between the two target terms “rich” and “poor” literally, and the attribute words to be used as reference points to measure the semantic similarity. It should be taken into account that the value obtained is a number between -1 and 1, since the cosine of an angle belongs to this interval. To carry out our study, we have applied the function arc cosine, presented in Eq. (3), to find the original value of the angle in its natural magnitude radians.

3 $$\uptheta =\mathrm{ arccosine}(\mathrm{similarity cosine})$$

#### Identifying logical relationships (analogies) in the same context (embedding)

A word embedding model can be evaluated on the basis of performance in solving analogy questions. This task was first introduced by Mikilov et al. (2013) and consists of performing additive operations between word vectors. The following equation summarises the so-called “analogy relation” that exists between vector operations.

4 $$\widehat{\mathbf{r}\mathbf{i}\mathbf{c}\mathbf{h}}-\widehat{\mathbf{w}\mathbf{o}\mathbf{r}\mathbf{d}1}=\widehat{\mathbf{p}\mathbf{o}\mathbf{o}\mathbf{r}}-\widehat{\mathbf{w}\mathbf{o}\mathbf{r}\mathbf{d}2}$$

Based on the above, one can seek to predict the vector of one of the words by clearing the equation as follows:

5 $$\widehat{\mathbf{w}\mathbf{o}\mathbf{r}\mathbf{d}2 }=\widehat{\mathbf{p}\mathbf{o}\mathbf{o}\mathbf{r}}+\widehat{\mathbf{w}\mathbf{o}\mathbf{r}\mathbf{d}1}-\widehat{\mathbf{r}\mathbf{i}\mathbf{c}\mathbf{h}}$$

The result of this equation would be the vector of the word2. In practice, cosine similarity is used to determine that the closest word vector corresponds to the correct answer of the analogy. As a result, we can provide evidence whether a word embedding model is able to maintain the semantic and syntactic relationship between words.

## Results and discussion

The proximity was calculated between the different attributes and the target terms “poor” and “rich”. In Table 1, the relative value of 1 indicates that the attribute is closer to “poor” than to “rich” in terms of cosine. Alternatively, relative distances can be calculated in radians and then results need to be read the other way round, namely, the longer the distance, the weaker the association between the attributes and the categories of rich and poor.

**Table 1:** Proximities and distances between unfavourable attributes and the key terms “poor” and “rich” and the ABI in Google News Word2vec pre-trained embeddings

Negative attributes	Proximity to “poor” (cosine)	Proximity to “rich” (cosine)	Relative value: 1 suggests attribute closer to “poor”	Relative distance to “poor” (in radians)	Relative distance to “rich” (in radians)	Aporophobia bias indicator (ABI)
Substandard	0.518799	0.065894	1	1.025350	1.504854	0.479503
Dreadful	0.496364	0.108623	1	1.051390	1.461958	0.410568
Mediocre	0.525181	0.157387	1	1.017868	1.412751	0.394883
Inferior	0.442338	0.154269	1	1.112590	1.415908	0.303316
Indifference	0.295424	0.049471	1	1.270896	1.521304	0.250408
Displeasure	0.181486	− 0.043921	1	1.388298	1.614732	0.226433
Humiliating	0.236273	0.013788	1	1.332267	1.557007	0.224740
Abhorrent	0.177211	− 0.034837	1	1.392643	1.605641	0.212997
Disgust	0.175618	− 0.033866	1	1.394262	1.604669	0.210406
Disrespect	0.178972	− 0.002676	1	1.390853	1.573472	0.182618
Disregard	0.165259	− 0.011534	1	1.404775	1.582331	0.177555
Fear	0.174980	0.019890	1	1.394910	1.550904	0.155994
Irritation	0.152907	0.011789	1	1.417287	1.559006	0.141719
Hostile	0.185884	0.045462	1	1.383824	1.525318	0.141493
Rudeness	0.176455	0.038615	1	1.393411	1.532171	0.138759
Annoyance	0.110991	− 0.026991	1	1.459575	1.597791	0.138215
Disgusting	0.259967	0.133528	1	1.307807	1.436867	0.129059
Hostility	0.132259	0.040978	1	1.438148	1.529806	0.091657
Rejection	0.100165	0.037907	1	1.470462	1.532879	0.062416
Contempt	0.091754	0.034602	1	1.478912	1.536186	0.057273
Hate	0.166657	0.111664	1	1.403357	1.458898	0.055540
Insult	0.150543	0.107800	1	1.419678	1.462786	0.043107
Aversion	0.169729	0.132875	1	1.400240	1.437526	0.037285
hate act	0.143041	0.111930	1	1.427262	1.458631	0.031369
hate speech	0.154789	0.134926	1	1.415381	1.435456	0.020075
Antipathy	0.082810	0.075422	1	1.487891	1.495302	0.007411

Source: author’s creation

The main advantage of using radians is that we can calculate “distances of distances” (DD), evaluating the difference between how a certain attribute is associated to “poor” as compared to “rich”, allowing a quantitative expression of the bias net effect, which we have named “aporophobia bias indicator” (ABI). The ABI, therefore, constitutes an intrinsic and preliminary way to evaluate bias against the poor in pretrained models for given attributes. We have named this model AWEAT (Aporophobia Word Embedding Association Test), since it is inspired on the WEAT (Word Embedding Association Test) by Caliskan et al. (2017).

The AWEAT allows to order and classify the different attributes from higher to lower ABI for a given pretrained embedding (Google News Word2Vec, Wikipedia GloVe and Twitter GloVe) and find out which negative attributes imply higher bias, since they are more closely related to the term “poor” as opposed to the term “rich”. If we consider that the lowest negative ABIs are around 0, 14 and that the highest are around 0, 5, we can split this interval into quartiles (following the standards of the Human Development Index). The cut-off points are less than 0.02 for low bias, 0.18 for medium bias, from 0.18 to 0.34 for high bias and above 0.34 to very high bias against the poor. This classification is based on the current selection of attributes. Should the attributes change, the classification should change accordingly.

This order and classification bring meaningful information to the research, since attributes, such as “antipathy”, “hate speech” and “hate act”, would be classified as low bias (in the sense of the level of association of these attributes to “poor” as compared to “rich” in Google News Word2vec pre-trained embedding), whereas at the other extreme, attributes, such as “mediocre”, “dreadful” and “substandard”, would be classified as very high bias. Therefore, we should distinguish here between association (distance) and gravity (seriousness) of a construct. In this analysis, we are not handling any evidence about the gravity of these attributes. Instead, our focus is on their degree of association (distance) with the poor in the characterisation of bias. For instance, as much as “substandard” seems to present the highest association with the term “poor”, as showed in Table 1, it seems to be a relatively inconsequential attribute if compared to “hate acts” or “insults” in terms of their gravity.

**Fig. 2:**
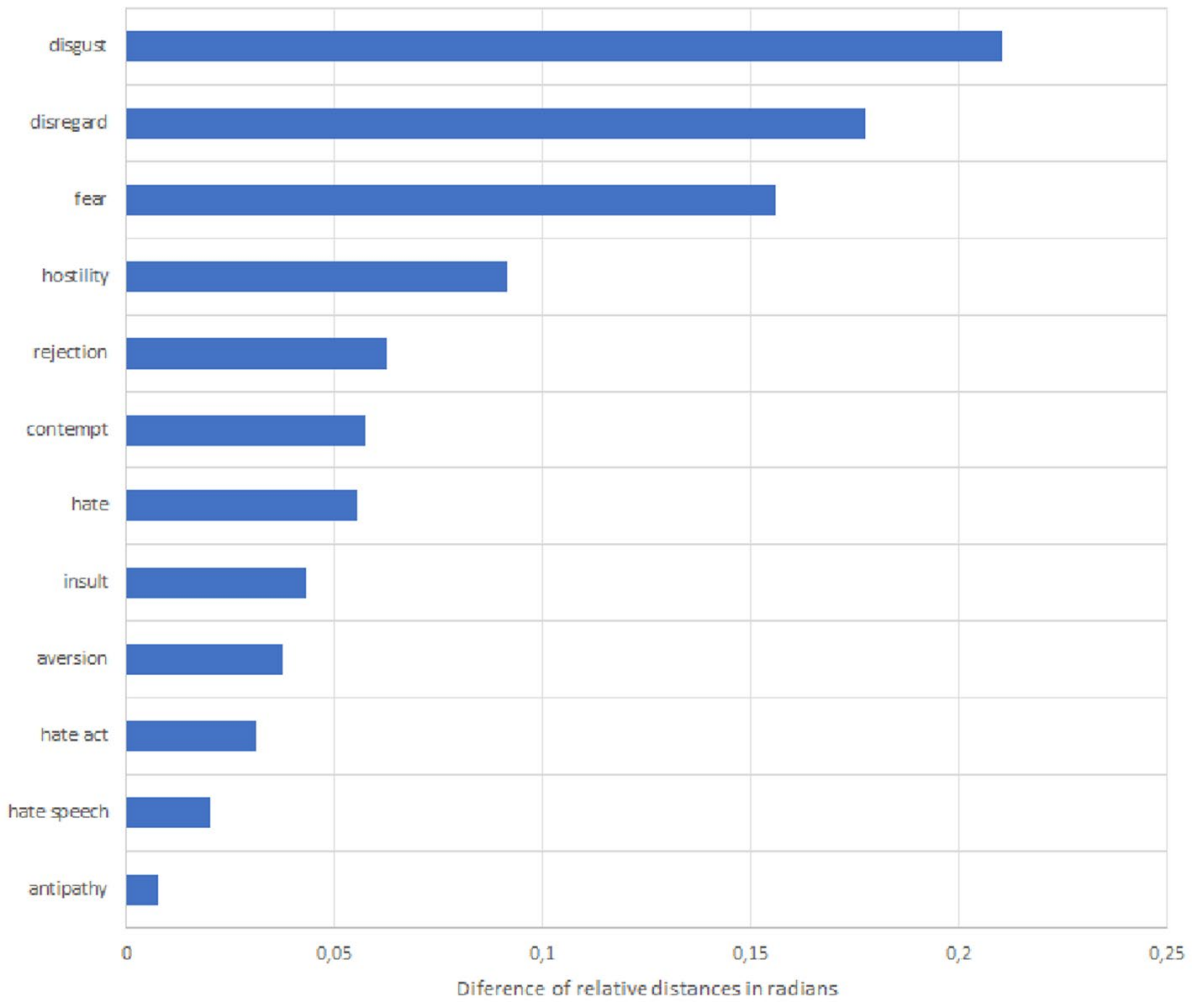
ABIs (difference in distance between how an attribute is associated to the term “poor” as compared to the term “rich”) for unfavourable attributes used by Cortina (2017) in Google News Word2vec pre-trained embeddings. Source: authors’ creation. OBS: These words have been used by Cortina (2017) and identified by Comim, Borsi and Valerio (2019)

It is also interesting to analyse some of the attributes that were originally used by Cortina (2017) to see how they compare to each other in terms of ABI. Although Cortina used them quite indistinctly in her discussion, it is possible to see from Fig. 2 that some attributes, such as ‘disgust’, ‘disregard’ and ‘fear’, appear to be more closely associated to the term “poor” (meaning that there is a lower relative distance of that attribute in relation to the term “poor” than in relation to the term “rich”) than others, such as ‘antipathy’ and ‘aversion’.

**Fig. 3:**
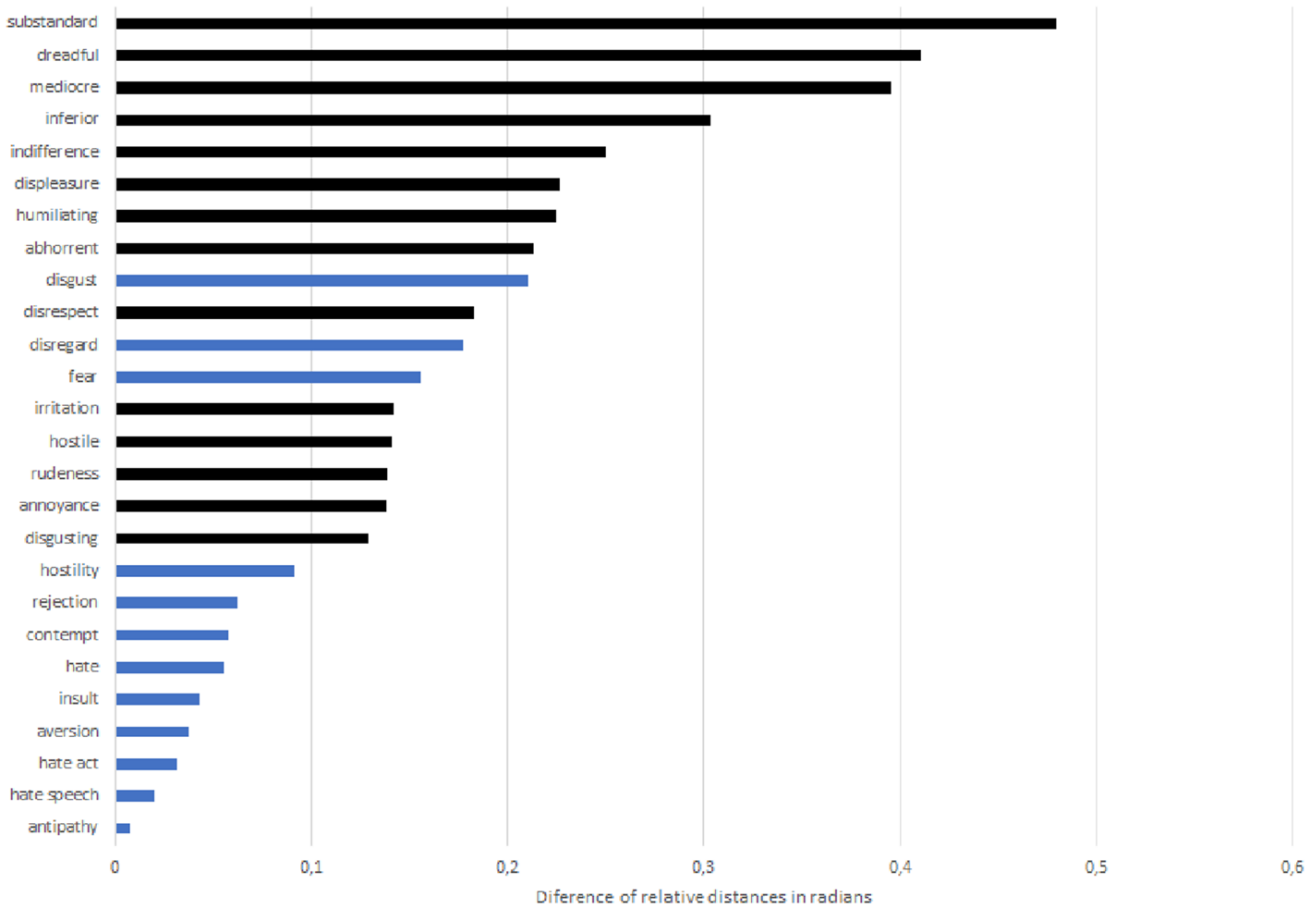
ABIs for unfavourable attributes in Google News Word2vec Pre-trained embedding. Unfavourable attributes used by Cortina (2017) are shown in blue. Source: authors’ creation

Our study, however, includes a wider range of negative expressions (other than those mentioned by Cortina) and this unveils a more complex reality. First, the range of attributes that are closely related to the term “poor” is much richer and more intense than the one originally used by Cortina. Figure 3 illustrates in blue the attributes used by Cortina and in black a sample of other attributes included in the study, following Allport’s categorization of prejudices according to the degree of associated action (Table 2 in the Appendix). As a result of broadening the semantic scope and the number of attributes, we find out that attributes that can be included under the categories of “beliefs” or “communication”, such as “substandard”, “mediocre” or “indifference”, according to Allport (1954), have clearly higher ABIs (Table 2). In contrast, attributes that have a stronger degree of action, such as “insult”, “hate speech” or “hate act”, which are associated to Allport’s categories of “discrimination” and “physical attack”, are more equidistant to the key terms “rich” and “poor” and therefore less closely associated to the poor.

When analysing the results of the favourable attributes (Table 3), two features are immediately evident from a first inspection. First, results for favourable attributes are not necessarily symmetric to unfavourable attributes (as expected, since the terms themselves are not completely symmetric). Second, some favourable attributes are more closely related to the term “poor” than to the term “rich”, characterising elements that *prima facie* could be understood as positive bias towards the poor. However, a close inspection reveals that attributes of “sympathy”, “politeness”, “pleasing”, “goodwill”, “cordiality” and “friendliness” are all compatible with a certain sense of subservience that can be expected from the poor, reinforcing a certain stereotype of inferiority. We can also verify that some words are relatively neutral towards the rich and the poor. On the other hand, the closer distances found out between favourable attributes and the “rich” reveal hedonist attributes related to attractiveness, pleasure, taste, etc., all part of elements of ‘distinction’, as famously portrayed by Bourdieu (2010). This phenomenon could be an evidence of plutofilia or overestimation of the rich, which, according to Allport is a previous step to aporophobia, since “one must first overestimate the things one love before one can underestimate their contraries” (1954: 25).

**Table 3:** Proximities and distances between favourable attributes and the key terms “poor” and “rich” and the ABI in Google News Word2vec pre-trained embeddings

Favourable attributes	Proximity to “poor” (cosine)	Proximity to “rich” (cosine)	Relative value: 1 suggests attribute closer to the poor	Relative distance to “poor” (in radians)	Relative distance to “rich” (in radians)	Aporophobia bias indicator (ABI)
Sympathy	0.169531	0.018321	1	1.400441	1.552474	0.152032
Politeness	0.132293	0.068439	1	1.438114	1.502303	0.064189
Pleasing	0.227241	0.174897	1	1.341551	1.394995	0.053443
Goodwill	0.088890	0.039868	1	1.481787	1.530918	0.049129
Cordiality	0.043623	0.007792	1	1.527159	1.563004	0.035845
Happy	0.212202	0.180576	1	1.356968	1.389223	0.032255
Fearless	0.100959	0.069186	1	1.469664	1.501554	0.031889
Pride	0.104457	0.088019	1	1.466148	1.482663	0.016514
Friendliness	0.178084	0.175157	1	1.391756	1.394731	0.002974
Courageous	1	1	0	0	0	0
Self-assurance	1	1	0	0	0	0
Carelessness	1	1	0	0	0	0
Defence	1	1	0	0	0	0
Affection	0.100301	0.10674	0	1.470325	1.463852	− 0.006474
Liked	0.125296	0.135883	0	1.445169	1.434491	− 0.010678
Delight	0.033640	0.045317	0	1.537149	1.525463	− 0.011687
Desire	0.085015	0.096916	0	1.485677	1.473728	− 0.011949
Pleasant	0.168783	0.187770	0	1.401201	1.381905	− 0.019297
acceptation	0.049464	0.099845	0	1.521311	1.470784	− 0.050527
appreciation	0.005268	0.075830	0	1.565527	1.494893	− 0.070635
independence	0.067198	0.141933	0	1.503546	1.428382	− 0.075165
Love	0.107482	0.184401	0	1.463105	1.385334	− 0.077772
Delightful	0.131124	0.215119	0	1.439293	1.353983	− 0.085311
Flattery	0.054658	0.140086	0	1.516110	1.430247	− 0.085864
Friendly	0.184168	0.271432	0	1.385570	1.295916	− 0.089655
Endorsement	− 0.049720	0.057279	0	1.620537	1.513486	− 0.107052
Taste	0.147377	0.261997	0	1.422879	1.305705	− 0.117175
Pleasure	− 0.005007	0.120311	0	1.575803	1.450193	− 0.125610
Attractive	0.146302	0.282672	0	1.423967	1.284217	− 0.139750

Source: author’s creation

**Fig. 4:**
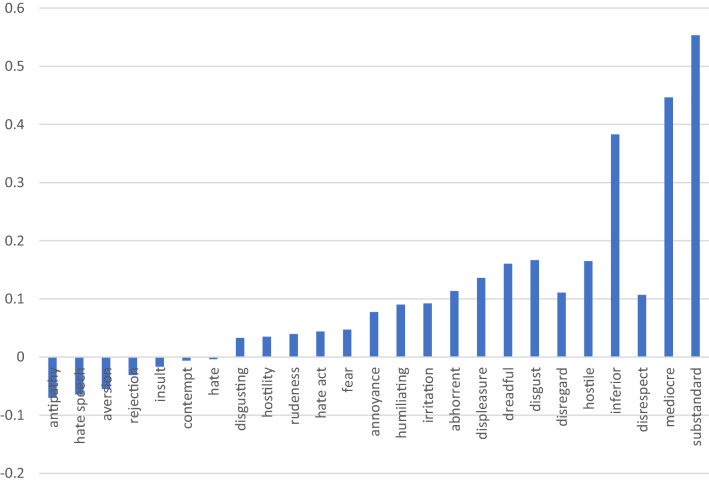
CABIs for unfavourable attributes in Google News Word2Vec vs Twitter GloVe, indicating the difference in the degree of bias per attribute between the two predefined embeddings. Source: authors’ creation

**Fig. 5:**
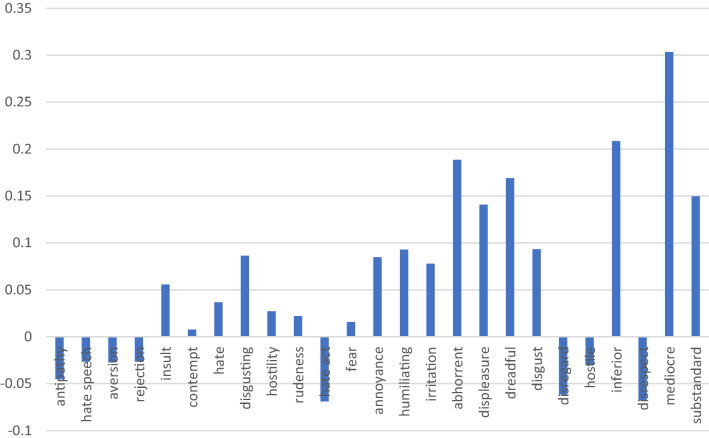
CABIs for unfavourable attributes in Google News vs Wikipedia, indicating the difference in the degree of bias per attribute between the two predefined embeddings. Source: author’s creation

**Fig. 6:**
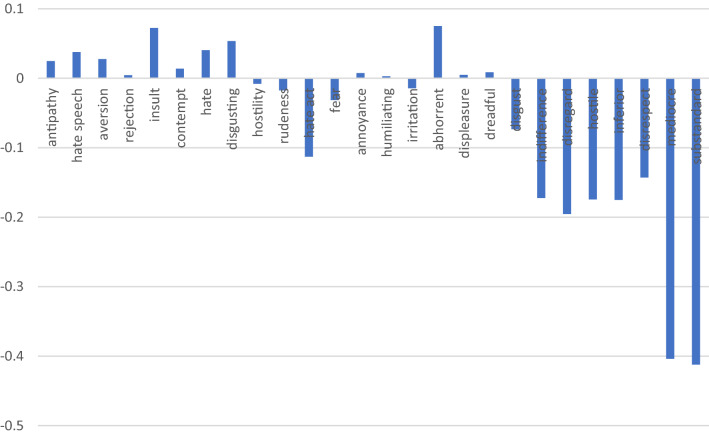
CABIs for unfavourable attributes in Twitter vs Wikipedia, indicating the difference in the degree of bias per attribute between the two predefined embeddings. Source: author’s creation

It is important to remark, however, that Google News Word2vec pre-trained embedding is not the only informational basis that has been used for this assessment. Two additional embeddings, trained on different databases, are integral part of the study, namely Twitter Glove and Wikipedia Glove. The coincidences between the three analysed embeddings provide robustness to the AWEAT model. Figures 4, 5 and 6 display the key results.

In Fig. 4, positive results indicate that the ABI in Google News is larger than the ABI in Twitter GloVe pretrained embedding. On the other hand, negative results uncover those attributes whose ABIs are higher in Twitter. In fact, by taking the difference between ABIs in the different embeddings, we are calculating a comparative ABI (CABI), resulting from the use of different informational bases, and we are able to see which embedding includes higher bias for specific attributes. In Fig. 4, evidence shows that for attributes related to Allport’s category of “belief” (see Table 2 in the appendix), such as “substandard”, “mediocre” or “inferior” the CABIs are positive, that is, the bias against the poor is relatively higher in Google News Word2Vec than in Twitter GloVe pretrained embeddings. This finding was unexpected in the study, since most sources in Google News are journalists and professionals (Bolukbasi et al. 2016), as compared to Twitter. Although more evidence is needed, this preliminary results could suggest that news could show higher bias against the poor, for the attributes that express beliefs.

On the other hand, negative CABIs suggest that bias against the poor is higher in Twitter GloVe, as compared to Google News Word2Vec, when the attributes correspond to Allport’s (1954) categories of “discrimination” or “physical attack” (see Table 2 in the Appendix), that is for attributes, such as “hate speech”, “aversion”, “rejection”, “insult” and “contempt”.

We find a similar trend, although not as consistent, when comparing the ABIs of unfavourable attributes between Google News Word2Vec and the Wikipedia Glove pre-trained embeddings (Fig. 5), suggesting that there is higher degree of bias against the poor in Google News in for attributes that express beliefs. When comparing Twitter GloVe and Wikipedia GloVe pre-trained embeddings (Fig. 6), bias expressed as actions under the categories “discrimination” and even “physical attack” (Table 2 in the Appendix) appears to be higher in Twitter, whereas bias expressed as beliefs is higher in Wikipedia or equidistant in the two pre-trained embeddings.

Finally, following Nadeem et al. (2020), we have calculated the distance between the key attributes “rich” and “poor” and neutral attributes using the names of plants, animals and planets, among other terms, to test the robustness of the AWEAT model. Although all terms show a bias (that is appear slightly closer to either “rich” or “poor”), only 4”neutral” terms out of 166 show an ABI level in the order of the first decimal. This proves, on the one hand, that we live in a market economy and therefore all terms have an economic association either to “rich” or “poor”. On the other, since this association is much lower than the “favourable” and “unfavourable” attributes used in the study, the test with “neutral” words validates the AWEAT model to evaluate bias against the poor in pre-trained embeddings by measuring the distances between “favourable” and “unfavourable” attributes associated to the poor as compared to the rich.

## Conclusion

This study offers a preliminary disruptive contribution to the body of work on bias with the first set of empirical data evidencing the existence of bias against the poor within the three pre-trained word embeddings included in the study, namely Google Word2Vec, Twitter and Wikipedia GloVe. As a result, this paper empirically illustrates a transversal type of bias that has been unnoticed, since it is an expression of fundamental shared values in welfare states: the belief of equal opportunity and individual responsibility to climb up the ladder. However, when this bias leads inevitably to discriminatory acts, it has serious consequences towards the achievement of the first Sustainable Goal of the United Nations (no poverty).

The article also provides evidence that there is a consistently higher degree of bias in Google News Word2Vec, as compared to the other two embeddings, when the attribute terms express beliefs and a higher level of bias against the poor in Twitter GloVe when the terms express behaviour. This preliminary results could suggest that some news in the media would express a higher level of bias against the poor than individuals in terms of expressed beliefs, whether individuals would offer a higher level of bias shown as behaviour (discrimination or physical attack), for the terms included in the study.

AI systems act as a warning flag of inconspicuous prejudices expressed as bias, but also contribute to spread biased opinions that can eventually lead to discriminatory behaviours. Further studies should be carried out with wider sample of target terms to mitigate the distorting effect of the polysemy of the selected terms “rich” and “poor”. It should also be analysed why, even when not referring to socioeconomic topics, “poor” has a negative connotation as compared to “rich”.

In addition, further studies could also include a wider list of attributes and pre-trained embeddings to obtain evidence on the impact of the bias against the poor on the communities that are historically disempowered as a result of other factors, such as gender, race, nationality or religion, to name some examples. A comparative study between the bias against the poor in Global North and the Global South would also be recommended, exploring the correlation between the bias against the poor in line with poverty and inequality levels as well as cultural factors. A deeper analysis is also required to compare biases through different social networks communication channels.

Although it is not possible to make the world a better place only through algorithms, they can contribute to make a diagnosis and monitor bias and discriminatory behaviours such as hate speech. This study, therefore, constitutes a first step towards taking action to mitigate pre-existing prejudices that can derive in discriminatory actions. In addition, this work constitutes an evidence for the need to oversee AI technologies and the opportunity that human-in-the-loop decision-making, the agreement on pro-ethical development and the implication of social science experts to analyse the roots of bias constitute to convert AI tools not only on autonomous reproducers (and often aggravators) of social inequalities, but on enables for sustainable development.

## Data Availability

The datasets analysed during the current study are included in this published article. Supplementary information files generated for the study are available from the corresponding author on reasonable request.

## Appendix

See Table 2.

**Table 2:** Terms included in the study categorized according to Allport’s (1957) degree of action associated to prejudice

	Favourable	Unfavourable	
Belief	superior, willpower, kind, courageous, calm, calmness, mildness, mild, innocuous, positive, dignified, delight, delightful, friend, friendship, courage, serenity, excellent, partner, pleasant, polite, brave, higher, adequate, true, happy, peace, peaceful, (28)	Belief	inferior, mediocre, negative, rude, rudeness, lower, shame, shameful, shameless, substandard, slight, carelessness, unkind, inoffensive, distaste, repugnant, rival, scared, sicken, upset, adversary, enemy, opponent (23)
Attitude & action:		Attitude & Action:	
Communication	acknowledgement, empathy, patience, tolerate, attentiveness, respectful speech, patience, cordiality, agreement, endorsement, attestation, regard, taste, remember, interest, tolerance, contentment, politeness, (19)	Antilocution	antipathy, disregard, no acknowledgement, denounce, denunciation, belligerence, belligerent, concern, denial, disagreement, derision, disregard, forget, ignore, indifference, absence of sympathy, refusal, defense, apathy, antagonism (20)
Acceptance	friendliness, friend, goodwill, kind, kindness, sympathy, acceptance, companionable, conciliate, fearless, cordiality, amicability, accord, self-assurance, attraction, desire, recommend, consonance, pleasure, pleasing, confidence, friendly, amity, affability, affection, benevolence, preservation, acquiescence, appetency, liking, becoming, pleasing, solace, love, love speech, liked, acceptance, accept, acceptation, like, complimentary, gentleness, attraction, attractive, approve, approval (46)	Avoidance	disgust, fear, impatience, afraid, alarmed, annoyance, annoying, anxiety, bitterness, challenger, corrupting, defense, defend, detestation, dislike, disgusting, disgust, disapprove, disapproval, detestation, displeasure, dread, dreadful, foe, ill feeling, ill will, irritating, irritation, loathe, loathing, opposition, repel, repugnance, repulse, repulsion, repulsive, resent, resentment, resistance, revulsion, unbecoming, undignified, upsetting, worry, calmness, independence, weighty, hate, abhorrence, abhorrent, hostile, hostility, neglect, unfriendliness, animosity, contempt (56)
Admiration	admiration, praise, approval, appreciation, delight, cherish, adore, flattery, pride, admirable, adulation, praise, dignified, appreciation, appreciate, respect, (16)	Discrimination	degrading, rejection, affront, anger, animosity, aversion, conflict, degrading, demeaning, disrespect, enmity, hatred, intolerance, obstruction, offense, offend, offensive, scorn, slur, shamed, unsupportive, hostility, abandonment, humiliating, hate speech, insult (27)
Aid	Aid, help, heal, support, love act, cooperation, comfort, facilitation, ally, shelter, encourage, encouraging (12)	Physical attack	hate act, physical aggression, abuse, abusive, aggression, assault, attack, bellicose, bellicosity, intimidate, intimidating, intimidation, violence, violent, harm, physical protection (16)

Original expressions used by Cortina (2017) appear underlined

Source: author’s creation
